# Gas Chromatography- Mass Spectrometry Based Metabolomic Approach for Optimization and Toxicity Evaluation of Earthworm Sub-Lethal Responses to Carbofuran

**DOI:** 10.1371/journal.pone.0081077

**Published:** 2013-12-04

**Authors:** Mohana Krishna Reddy Mudiam, Ratnasekhar Ch, Prem Narain Saxena

**Affiliations:** 1 Analytical Chemistry Section, CSIR-Indian Institute of Toxicology Research, Lucknow, Uttar Pradesh, India; 2 Academy of Scientific and Innovative Research, CSIR-Indian Institute of Toxicology Research, Lucknow, Uttar Pradesh, India; 3 SEM Facility, CSIR-Indian Institute of Toxicology Research, Lucknow, Uttar Pradesh, India; Imperial College London, United Kingdom

## Abstract

Despite recent advances in understanding mechanism of toxicity, the development of biomarkers (biochemicals that vary significantly with exposure to chemicals) for pesticides and environmental contaminants exposure is still a challenging task. Carbofuran is one of the most commonly used pesticides in agriculture and said to be most toxic carbamate pesticide. It is necessary to identify the biochemicals that can vary significantly after carbofuran exposure on earthworms which will help to assess the soil ecotoxicity. Initially, we have optimized the extraction conditions which are suitable for high-throughput gas chromatography mass spectrometry (GC-MS) based metabolomics for the tissue of earthworm, *Metaphire posthuma*. Upon evaluation of five different extraction solvent systems, 80% methanol was found to have good extraction efficiency based on the yields of metabolites, multivariate analysis, total number of peaks and reproducibility of metabolites. Later the toxicity evaluation was performed to characterize the tissue specific metabolomic perturbation of earthworm, *Metaphire posthuma* after exposure to carbofuran at three different concentration levels (0.15, 0.3 and 0.6 mg/kg of soil). Seventeen metabolites, contributing to the best classification performance of highest dose dependent carbofuran exposed earthworms from healthy controls were identified. This study suggests that GC-MS based metabolomic approach was precise and sensitive to measure the earthworm responses to carbofuran exposure in soil, and can be used as a promising tool for environmental eco-toxicological studies.

## Introduction

Metabolomics, an omics science of systems biology, is the untargeted profiling of endogenous metabolites within a biological system under various physiological conditions and offers a unique opportunity to look at genotype-phenotype relationships as well as genotype-environmental relationships [1], [2]. Metabolomic profiling provides a powerful approach to identify and to quantitatively measure global changes in metabolites from biochemical pathways that are altered in response to disease, therapeutic intervention or toxicant. It has been widely employed in functional genomics, disease diagnosis, clinical [3], food and nutritional science [4], toxicology and pharmacology research [5]. Environmental metabolomics is an emerging field of science that provides wide opportunity to characterize the interactions of living organisms with the environment for assessing the organism functions at a molecular level, which will significantly contribute to the environment and human health [6], [7], [8], [9]. Recent metabolomic studies have identified novel biomarker patterns in terrestrial invertebrates [10], [11], [12], [13]. Earthworms, the soil engineers are a key terrestrial organism species and perform a number of essential functions like decomposition of organic matter, tillage and alteration of the soil and enhancement of microbial activity. Environmental pollutants, including organic chemicals and toxic metals may induce variety of adverse effects on ecosystems [14]. Indirectly, these effects of organic pollutants and metals are monitored by taking the effects on earthworms as an illustration [15]. Traditionally, a broad group of biomarkers such as cholinesterase (ChEs), DNA breakage, cytochorme P-450-dependent mono oxygenases, enzymes of oxidative stress have been used as biomarkers for heavy metal (Zn, Pb, Cd, Cu) and organic pollutant exposure by taking earthworm as a model organism for both laboratory and soil representative studies [16]. Over the past decade, varieties of endogenous metabolites have been identified as potential biomarkers of different environmental chemicals exposure to earthworms, for e.g. decrease in lactate and fatty acids for poly aromatic hydrocarbon (PAH) pyrene exposure [17], increase in alanine for pesticides DDT & endosulfan exposure [18], increase in histidine for copper exposure [19].

Metabolomics cover a vast range of metabolites and aim to determine the changes in low molecular weight organic metabolites in complex biological matrices. High-field proton nuclear magnetic resonance (^1^H NMR) spectroscopy and mass spectrometry (MS) based techniques are the major analytical tools used for metabolomic studies. NMR offers advantages of minimal sample preparation and quantitative measurement of metabolites. However, only most abundant peaks were observed due to its limited sensitivity with a detection threshold of 5 nmol and limited dynamic range. Extensive overlapping of signals in most of regions, especially carbohydrate and lipid regions of NMR spectrum makes difficult in metabolomic studies [20], [21]. Compared to NMR, MS methods are sensitive and can serve as a stand-alone method for identifying compounds from complex mixtures. The combination of mass spectrometry with gas chromatography has become a powerful metabolomic tool, provides high chromatographic resolution, reliable and reproducible mass fragmentation pattern of metabolites with large dynamic range, as well as the capability to identify unknown compounds for global metabolomic profiling of intracellular metabolites [22]. These advantages make gas chromatography-mass spectrometry (GC-MS) widely acceptable analytical tool for environmental metabolomic studies [23], [24], [25], [26], [27], [28].

Sample preparation strategy plays an important role in the metabolomic profiling. Metabolism quenching, tissue disruption and extraction were the key steps for metabolomic profiling. Various sample preparation protocols have been developed for species specific metabolome profile such as bacterial cells [29], different mammalian cells [30], [31], [32], microorganisms [33], *Caenorhabditis elegans* [34], ragworms [35], and different human matrices such as blood [36], urine [37], CSF [38], and human faecal water [39]. An optimal extraction method should obey the criterion, which includes extraction of large number of metabolites without affecting their stability, easily adaptable to analytical technique, and should be reliable and reproducible in order to identify metabolomic changes [40], [41]. Although previous studies for the optimization of tissue extraction protocols for non-targeted metabolomics of earthworm based on four solvent systems have been reported using different analytical platforms, but not been extensively studied using GC-MS based metabolomic platform [42] [43]. In the present communication, an attempt has been made to standardize the extraction conditions to explore the performance of non-targeted GC-MS based metabolomic approach for earthworm and applied to evaluate the toxicity of carbofuran in earthworm, *Metaphire posthuma*.

Carbofuran (2, 3-dihydro-2, 2-dimethyl-7-benzofuranyl-N-methyl carbamate) is a potent broad spectrum systemic insecticide, nematocide and acaricide commonly used for agriculture purposes [44], [45]. As a result of its wide spread use, it has been detected in surface and ground water [46]. European Union commission and United States EPA have considered that, it is very toxic to mammals, aquatic organisms and invertebrates [47]. It is having miticide activity, which acts by surface contact and through ingestion by interfering with the transmission of nerve impulses by inhibiting cholinesterase [48], [49]. It causes reversible acetyl cholinesterase carbamylation and allowing the accumulation of acetylcholine. Its application was found to reduce the total number of earthworms by 83% and the total biomass by 60% [50], [51].

Non-targeted GC-MS based metabolomic approaches can be subjected to supervised analysis methods in order to classify toxicity patterns or metabolic trajectories associated with carbofuran induced metabolomic changes. For this, initially we have optimized extraction solvent for global metabolomic profiling of earthworm *Metaphire posthuma*. *Metaphire posthuma* is a soil dwelling species and it is widely found in agriculture field. Due to this advantage we want to explore the metabolic responses to this species, which in turn can help to evaluate soil ecotoxicity. Previous studies have been conducted on other species at laboratory conditions [52], [53], [54], [55]. After that, we have investigated the metabolomic profiles for carbofuran induced toxicity evaluation, using non-targeted GC-MS based metabolomic approach combined with pattern recognition methods. Three different concentrations 0.6 mgkg^−1^, 0.3 mg kg^−1^ and 0.15 mg kg^−1^ corresponding to their LC_50_ of 1/20, 1/40, 1/80 are exposed by spiking in soil, in addition to an unspiked soil control [50], [51]. Metabolomic perturbations were identified in carbofuran exposed samples from healthy controls. Metabolomic approaches can be used in the investigation of the unique mode of action and ecotoxicological risk assessment of bioactive compounds [56], [57].

## Materials and Methods

### Chemicals and Reagents

All chemicals used were analytical grade unless otherwise stated. Methoxyamine hydrochloride, carbofuran and N-methyl-N-trimethylsilyl trifluoroacetamide (MSTFA) and all standards of amino acids, sugars and organic acids were procured from Sigma-Aldrich (St. Louis, MO, USA). Solvents like chloroform, acetonitrile, methanol (MeOH) and iso-propanol were obtained from Sigma-Aldrich (St. Louis, MO, USA). Pellet pestle with a cordless motor was procured from Sigma Aldrich (St. Louis, MO, USA). The ultra pure water was prepared by RiOsTM water purification system (Millipore, Billerica, MA, USA). IMECO ULTRA SONICS (Bombay, India) was used as sonicator. Heto GD-2 maxi dry plus was used as a lyophilizer.

### Tissue disruption and extraction of metabolites

Earthworms were flash frozen in liquid nitrogen and stored at -80°C until further use. Earthworms were grounded to powder using a mortar and pestle in presence of liquid N_2_ in order to prepare a single homogenate sample. The homogenate was divided into three equal aliquots of 100 mg in a 2 ml of eppendorf tube. These tissue samples were extracted with different solvent systems such as methanol/chloroform/water (MCW; 2∶1∶1, *v/v/v*), methanol/water (MW; 4∶1, *v/v*), Acetonitrile/methanol/water (AMW; 2∶2∶1, *v/v/v*), pure methanol (MeOH), methanol/isopropanol/water (MIPW; 5∶2∶2, *v/v/v*) with a volume of 600 µl of each solvent system at 0°C. The resultant mixture was centrifuged at 10000 rpm for 3 min at 4°C. The procedure was repeated about three times.

### Derivatization, GC-MS analysis and spectral acquisition

Tissue extracts were lyophilized and added 90 µL of O-methoxyamine hydrochloride (20 mg mL^−1^) solution in anhydrous pyridine to the dried extracts. The resultant mixture was mixed vigorously using cylcomixer for one min and incubated for 30 min at 60°C in a heating block. Subsequently, 200 µL of MSTFA with 1% TMCS as catalyst was added and the extracts were incubated at 60°C for further 60 min. Analyses were performed using Trace GC gas chromatograph coupled with Quantum XLS mass spectrometer (Thermo Scientific, FL, USA). The injector, ion source and transfer line temperatures were set at 250°C, 220°C and 290°C respectively. The initial oven temperature was held at 65°C for 2 min, increased to 230°C at a rate of 6°C/min and finally increased to 290°C at a rate of 10°C/min (held for 20 min). Helium was used as a carrier gas at a flow rate of 1.1 ml min^−1^. An aliquot of 1 µL of the extracts were injected into the TG-5 MS capillary column (30 m x 0.25 mm i.d. x 0.25 µm film thickness) consisting of a stationary phase of 5% phenyl 95% methyl polysiloxane in the split less mode. Detection was achieved using mass spectrometer in electron impact ionization at 70 eV. Full scan mass spectra was acquired in the mass range of 45–750 Da at 1 scans s^−1^ rate with an initial solvent delay of 6 min.

### Data Pre-processing

All samples used for metabolomic profiling were analyzed as a single batch in a random order to minimize any analytical error, subjective interference and to keep the minimum retention shift. Chromatogram acquisition and data handling were carried out by using the GC-MS Xcalibur software (Thermo scientific, FL, USA). AMDIS software (Automated Mass Spectral Deconvolution and Identification System, version 2.0) was used to identify the metabolites in chromatographs. The mass spectra of all the detected compounds were compared with spectra in National Institute of Standards and Technology (NIST) library (version 2.0) or standards for confirmation. Baseline correction, noise reduction, smoothing and integration were performed using Thermo XCalibur software. Peaks resulting from the column bleeding and reagent peaks were excluded from the analysis. Blank experiments were also performed in order to minimize possible sources of contamination such as reagent impurities, contamination during sample preparation and any instrumental contamination. Intensity of target ion was used for peak integration due to its higher specificity, precision and identity [22], [58]. Only peaks shown higher reproducibility were taken for data analysis. Only those metabolites which have shown similarity index (SI) greater than 70% during spectral search using NIST library were taken into consideration for further analysis.

### Statistical analysis

The GC/MS data matrix for metabolomic analysis was composed of the composition of metabolites (columns) and samples (rows). Data were mean-centred and unit variance scaled to remove the offsets and adjust the importance of low and high abundance metabolites to an equal level. The resulting scaled data were imported to Statistica (version 10, Statsoft, Tulsa, OK) and Metaboanalyst (version 2.0) [59], [60], for statistical analysis. Heat map combined with two-dimensional hierarchical cluster analysis (2D-HCA) for the visualization of the metabolomics data were created using the software PermutMatrix version 1.9.3 (http://www.lirmm.fr/~caraux/PermutMatrix/) [61]. Principal component analysis (PCA) was performed to explore the clustering behaviour of the metabolites/samples. To identify the differential metabolites that account for the separation between groups, Partial least square discriminate analysis (PLS-DA) was applied. PLS-DA model was validated using the leave one out cross validation method and the quality of model is assessed on R2 and Q^2^ scores [62], [63]. Excellent models are obtained when the cumulative values of R^2^Y and Q^2^Y are above 0.8 [64]. In addition to cross validation, model validation was also performed by 500 times permutation tests [65]. Variable importance in projection (VIP) scores was obtained from the PLS-DAs. VIP scores are weighed as sum of scores of the PLS loadings. VIP scores give the information of the relative contributions of individual metabolites to the variance between the control and exposed groups. The higher the VIP value, the greater is the individual contribution of that metabolite to group separation. Metabolites with variable importance in projection (VIP) values of greater than 1 were identified as potential marker metabolites [66], [67], [68]. Univariant analysis was performed to these metabolites by applying unpaired *t-test* with a significance value (*p*) less than 0.05. Classification performance of biomarkers was further evaluated with receiver operator characteristic curve (ROC) based on support vector machine (SVM). The area under the receiver operating characteristic curve (AUC) was used as a measure of classification performance. The AUC with value 0.9–1.0 considered as excellent classification performance [69]. ROC curve analysis was performed by using freely available web based software ROCCET (http://www.roccet.ca).

### Soil spiking and earthworm exposure

The experimental procedure was adopted from previously reported procedure [70], [71], briefly, the procedure was as follows. The test worms (adult *Metaphire posthuma*) were collected from culture and placed in soil to acclimatize for seven days in a BOD incubator (Indian Equipment Corporation, Bombay, India) at 20±1°C. Before acclimatization soil composition was determined. The constituents of soil and pH were measured and found to be pH 7.1, Nitrogen 64 mg/kg, Phosphorus 8.3 mg/kg, Potassium 28 mg/kg, Magnesium 36 mg/kg, sand 87% [72]. Initially, four soil treatments were prepared including control. One kg of each soil was placed in three different trays and spiked with concentrations of 0.15, 0.3 and 0.6 mg kg^−1^ of carbofuran in equal volumes of acetone. Soil samples were then mixed well and vented for three hours to remove all the solvent. Then the soil samples were wetted with water to bring the soil to 50% of field capacity and then left to stabilize for 24 hours prior to addition of worms. Carbofuran spiked soil samples were transferred into l litre jars. An unspiked control treatment was prepared in the similar way. The adult earthworms with visible clitellum were collected from culture, weighed (average weight, 1.55±0.21 g). One kg of soil was used for six replicates per dose. Earth worms were transferred into individual jars and kept in dark during the exposure period as per Organisation for Economic Cooperation and Development (OECD) 1984 guidelines [71]. We did not observe the earthworms on the surface of the soil throughout the experimental period. After seven days of exposure, worms were weighed (average weight 1.20±0.16 g) and washed with distilled water to remove residual soil deposited on the surface of the earthworms. After washing with distilled water, earthworms were allowed to depurate for 15 hours on a filter paper and placed into a glass beaker to release their gut contents.

## Results and Discussion

**Figure 1:**
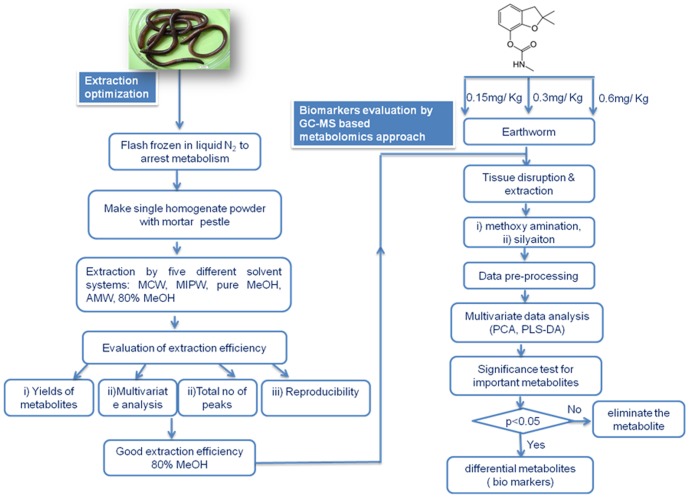
Workflow for the optimization and evaluation of earthworm responses to sub-lethal toxicity of Carbofuran.

The workflow strategy was shown in fig 1 consisting of optimization of extraction conditions followed by biomarker evaluation of carbofuran exposure to earthworms.

### Optimization of extraction solvent system

Ideally, an extraction solvent should obey the following properties i) it has to cover a broad range of chemical properties of metabolites to enable extraction of all metabolites in high yields with good reproducibility [43], ii) solvent system should not affect the stability of metabolites extracted [42]. Since each extraction solvent has its own chemical and physical properties, it is not easy to generate such an extraction solvent for global metabolomic profiling of earthworms. To select an optimal extraction solvent system for the global metabolomic profiling of earthworm, *Metaphire posthuma*, we have evaluated five different solvent systems, MCW, 80% MeOH, MIPW, AMW and pure MeOH, which are known to have good extraction efficiency for various tissue and cell metabolome [30], [31], [32], [33], [34], [35]. Out of the five solvents tested, best extraction solvent was selected based on (i) peak intensity of structurally identified metabolites, (ii) multi variant analysis and (iii) distributions over individual metabolite features of coefficient of variation (reproducibility) [30], [34], [36], [73]. The extraction results in various compounds from the tissue of earthworm which includes, amino acids, organic acids, carbohydrates, fatty acids, phosphates, polyols and amines (table 1).

**Table 1:** Compounds structurally identified from earthworm, *Metaphire.posthuma* by GC-MS.

Rt (min)	compound	fragmentations (m/z)	Idn	Rt (min)	compound	fragmentations (m/z)	Idn
	Amino acids & their derivatives				Fatty acids		
9.16	L-alanine	116,73,147,190,59	A	21.41	Tridecanoic acid	117,73,271,129,145	A
9.48	L-glycine	102,73,147,75,204	A	21.54	Tetradecanoic acid	285,117,75,129,145	A
11.53	L-valine	144,73,218,145,100	A	19.03	Tri decanoic acid methyl ester	74,87,55,143,185	A
12.23	L-leucine	158,73,147,102,59	A	19.58	Dodecanoicacid	75,117,257,132,145	A
12.34	L-isoleucine	158,73,218,147,232	A	26.98	Hexadecanoic acid	117,313,73,129,145	A
14.70	L-serine	204,73,218,147,100	A	27.71	9,12-octa deca dienoic acid (Z,Z) methyl ester	67,81,95,55,109	B
15.29	L-threonine	73,117,218,147,101	A	27.80	9-Octadecenoic acid (Z)methyl ester	55,74,69,83,97	B
17.90	L-methionine	176,128,73,61,147	A	27.90	11-octadecenoic acid (E) methyl ester	55,69,74,83,97	B
17.75	L-aspartic acid	73,232,100,147,218	A	28.24	Octadecanoic acid methyl ester	74,87,143,298,255	B
19.87	L-glutamic acid	246,73,128,147,156	A	28.46	Heptadecanoic acid	73,117,327,132,145	A
17.82	L-lysine	84,73,156,102,128	A	28.98	6-Hexadecenoic acid-7-methyl ester (Z)	138,55,69,97,83	B
18.47	L-phenylalanine	218,192,73,91,147	A	29.45	9,12-octa deca dienoic acid (Z,Z)	75,81,67,129,337	B
22.23	L-ornithine	174,73,142,186,348	A	29.61	11-Cis octadecenoic acid	339,73,117,129,55	B
24.43	L-tyrosine	218,73,280,100,147	A	29.94	Octadecanoic acid	117,341,73,132,145	B
23.94	N-α-acetyl-L- lysine	174,73,156,86,59	B	30.16	8,11,14-Eicosa trienoic acid mehtyl ester (Z,Z,Z)	79,67,93,55,150	A
18.09	pyroglutamic acid	156,73,147,230,258	B	30.24	5,8,11,14-Eicosa tetraenoic acid methyl ester(all Z)	79,91,67,105,55	B
	Organic acids			30.37	5,8,11,14,17-Eicosa pentaenoic acid methyl ester(all Z)	79,91,67,105,119	B
8.09	pyruvic acid	73,174,45,89,59	A	30.62	Cis-13-Eicosenoic acid methyl ester	55,69,74,83,97,297	A
13.59	scuccinic acid	147,73,247,129,45	A	31.47	Arachidonic acid	73,91,67,117,55	A
14.29	2-butenedioic acid	245,147,73,45,83	B	31.55	Cis-5,8,11,14,17-Eicosapenta enoic acid	79,73,91,117,67	B
16.43	malonic acid	73,147,305,45,69	A	31.70	α-Linoleic acid	73,79,67,95,55	A
17.44	malic acid	73,147,233,245,133	A	32.45	Cis-7,10,13,16-Docosa tetra enoic acid methyl ester	79,91,67,105,55	B
18.07	2-ketoisovaleric acid	73,147,157,232,260	B	33.45	2-mono palmitin	129,218,73,147,103	B
19.08	glutaric acid	73,147,198,156,288	B	33.76	Eicosanoic acid glycerate ester	73,57,147,43,129	B
11.01	2-butenoic acid	147,73,231,45,66	B	33.67	Hexadecanoic acid glycerate ester	371,460,239,73,147	B
					polyols (poly hydric alcohols)		
8.26	lactic acid	147,117,73,191,133	A	12.78	glycerol	73,147,205,117,103	A
19.05	2-hydroxy glutaric acid	73,129,147,247,349	B	27.19	Inositol	73,318,147,217,305	A
26.72	d-(+)gluconicacid	73,319,147,129,220	B	28.15	myo Inositol	73,217,147,305,191	A
	Carbohydrates			21.22	threitol	73,147,103,205,217	B
24.31	fructose	73,103,217,307,147	A	25.65	mannitol	73,319,147,205,217	A
25.30	glucose	73,319,205,147,218	A	31.38	1-O-pentadecylglycerol	205,147,73,117,131	B
25.16	mannose	73,147,319,205,160	A	32.78	1-O-hexadecylglycerol	205,147,117,73,133	B
25.47	Galactose	73,205,319,147,217	A		Phosphates		
34.22	turanose	73,361,147,217,103	B	12.98	phosphoric acid	299,73,133,314,211	B
35.02	lactose	204,73,191,217,361	A	10.78	methyl phosphate	241,73,133,256,211	B
35.51	maltose	204,73,191,361,217	A	22.80	1-glycero phosphate	357,73,299,147,103	B
34.79	sucrose	361,73,217,147,271	A	22.96	O-phospho ethanolamine	73,299,188,174,315	B
35.69	melibiose	204,73,191,217,129	B		galactose-6-phosphate	73,299,387,315,147	B
26.47	talose	204,73,191,217,147	B		amines		
24.85	methyl mannopyranoside		B	21.95	cadaverine	174,73,86,59,100	B
	Others			9.59	ethanolamine	174,73,147,100,86	B
39.31	cholesterol	129,329,368,73,353	A	34.57	N-acetyl glucosamine	73,147,103,205,129	B
36.50	cholesta-3,5-diene	368,147,105,91,145	B	14.67	2-amino-3-phenyl propane	188,73,100,147,114	B
25.91	Indole acetic acid	202,319,73,188,154	B				

Rt  =  retention time, Idn  =  identification, A  =  These compounds are confirmed by authentic standards, B  =  These compounds are identified by NIST library Mass Spectral fragmentation pattern.

For an unbiased analysis of metabolome, it is important to extract all the existing metabolites in high yields, including those metabolites present in relatively low concentrations. So as a first criterion, peak intensities of representative intracellular metabolites from various chemical groups, including amino acids, organic acids, carbohydrates, fatty acids, polyhydric compounds, phosphates and amines were compared by using five different solvent extraction systems. Fig S1 shows the extraction efficiency of these solvents in terms of total intensity over a wide range of chemical classes. Fig S1A shows that highest yields for amino acids were obtained using AMW as extraction solvent, which is similar to the earlier reported study on earthworm species *Lumbricus rubellus*. Highest extraction efficiencies of carbohydrates were obtained with MIPW and 80% MeOH as compared to pure methanol (Fig S1B). It may be due to high polar nature of sugars (poly hydroxyl compounds) and their solubility in solvents with high polarity, like MIPW and 80% MeOH as compared to less polar solvent MeOH. In case of fatty acids, pure MeOH has shown the highest extraction efficiency followed by 80% MeOH as depicted in Fig S1C. Organic acids, phosphates and polyols exhibited highest peak intensities when 80% MeOH was used as an extraction solvent depicted in Fig S1D-F.

**Figure 2:**
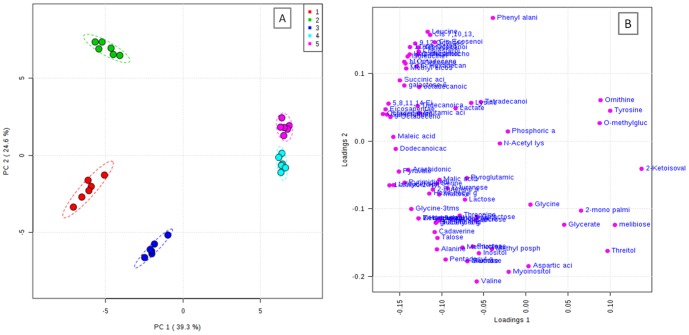
PCA of optimization of extraction solvent system for global metabolite profiling of earthworm *Metaphire posthuma* A) scores plot, explaining the extraction efficiency of different solvent systems 1. 80% MeOH, 2. Pure MeOH, 3. MIPW, 4. MCW, 5. AMW. B) Loadings plot.

Multivariate analysis was used to provide visualization of the overall affects of different solvent systems on the metabolomic profiles of earthworms [34], [35], [36]. PCA model has been constructed to obtain the overview of comparison of metabolic profiles in different solvent systems. The PCA model with R^2^X = 0.639, Q^2^X = 0.507 has been obtained based on cumulative values up to PC 2 shown in Fig 2. From this model, it was evident that, there were clear differences between the solvent systems in the extraction profile of metabolites. Score plot reflects the contribution of each principal component of all the solvent systems used for extraction and loading plot reflects the importance of the weight of metabolites. Based on the score plot, extraction of metabolites with 80% MeOH, MIPW and pure MeOH were shown completely different pattern of extraction than from the extraction with AMW, MCW. On the basis of differential distribution of metabolites on the bi plot, most of the metabolites significantly extracted into solvent systems like 80% MeOH, MIPW and pure MeOH, which were clustered to one side of PC1 (Fig S2), which is similar to the trend observed in metabolite peak intensities as shown in Fig. S1.

Overall 325 peaks were detected by GC-MS in that 84–91 peaks were identified and other 136–233 peaks were remain unidentified in all five extraction solvent systems. Maximum numbers of peaks were detected by 80% MeOH and MIPW with 325 and 317 peaks respectively. In the same way maximum number of identified peaks was detected in 80% MeOH and MIPW respectively. The total number of identified and unidentified peaks was depicted in table S1. Fewer numbers of peaks were detected and identified in Pure MeOH and MCW.

**Figure 3:**
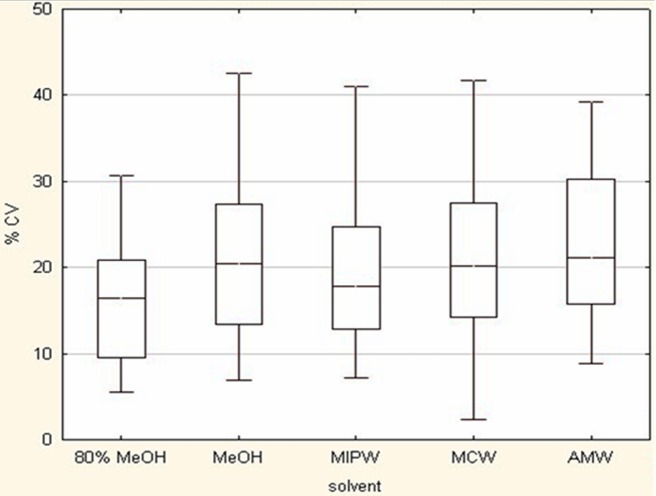
Coefficient of variation of metabolite features for different solvent systems extracted from earthworm *Metaphire posthuma*.

Reproducibility is another criterion for the selection of optimal extraction solvent and it is an important analytical parameter to assess the efficiency of extraction solvent. The box plot in Fig. 3 shows the distribution of coefficient of variation of identified metabolites for different extraction solvent systems. The median %CV values of identified metabolites for 80% MeOH, pure MeOH, MIPW, MCW and AMW were found to be 16.45, 20.39, 17.76, 20.12 and 21.14 respectively. Highest reproducibility was shown by 80% MeOH and after that MIPW. Previous methods showed a median CV values in the range of 15–30% across all analytical platforms. The present results coincide with the previously reported extraction protocols [28].

**Figure 4:**
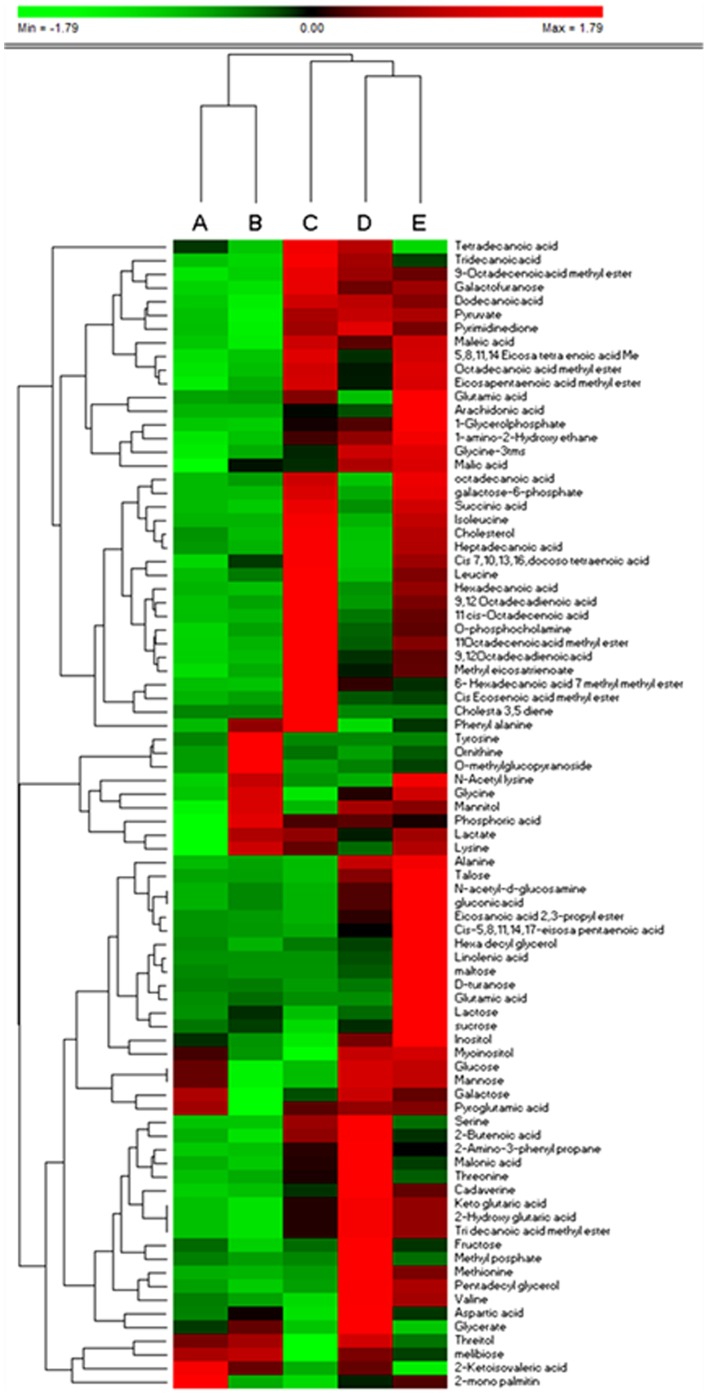
Clustered heat map of intracellular metabolites extracted using A) MCW B) AMW C) Pure MeOH D) MIPW E) 80% MeOH.

Hierarchical clustering was performed to identify significant differences in the extraction of metabolites using different solvent systems. The Hierarchical clustering analysis for the identified metabolites was depicted in Fig 4. The metabolites were placed in rows, and the sum of the peak intensities were normalized by employing unit variance. Ward's algorithm was used for this Hierarchical clustering. It clearly clustered the solvent systems, 80% MeOH, pure methanol and MIPW as one group, MCW and AMW as the other group with 80% MeOH producing the highest yield with respect to recovery. Very less recovery was observed with MCW. The results of Hierarchical clustering analysis were very well correlated with that of the multivariate analysis in terms of clustering and extraction efficiency of 80% methanol. The GC-MS chromatograph for the optimized extracted solvents for *M.posthuma* was shown in Fig S3. Overall, 80% methanol was found to be an optimal extraction solvent system based up on the criteria like highest peak intensity, multivariate analysis, reproducibility and hierarchical clustering with good extraction efficiency.

### Evaluation of carbofuran toxicity on earthworms by metabolomic approach

**Figure 5:**
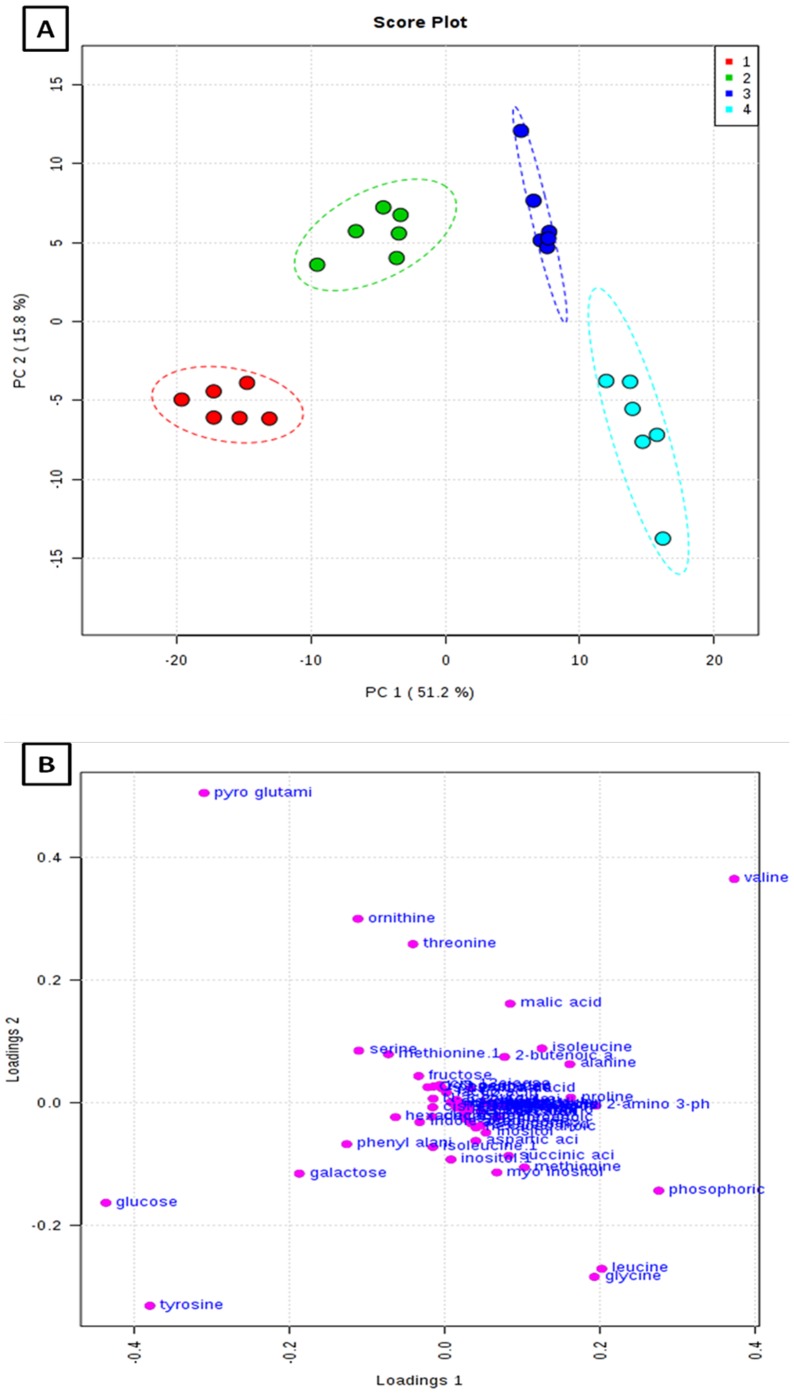
Principle component analysis (PCA) A) scores and B) loadings plots for 1.control earthworms and earthworms exposed to soil spiked with 2. Concentration of 0.1 mg/Kg, 3. Concentration of 0.3 mg/Kg, and 4. Concentration of 0.6 mg/Kg of carbofuran.

**Figure 6:**
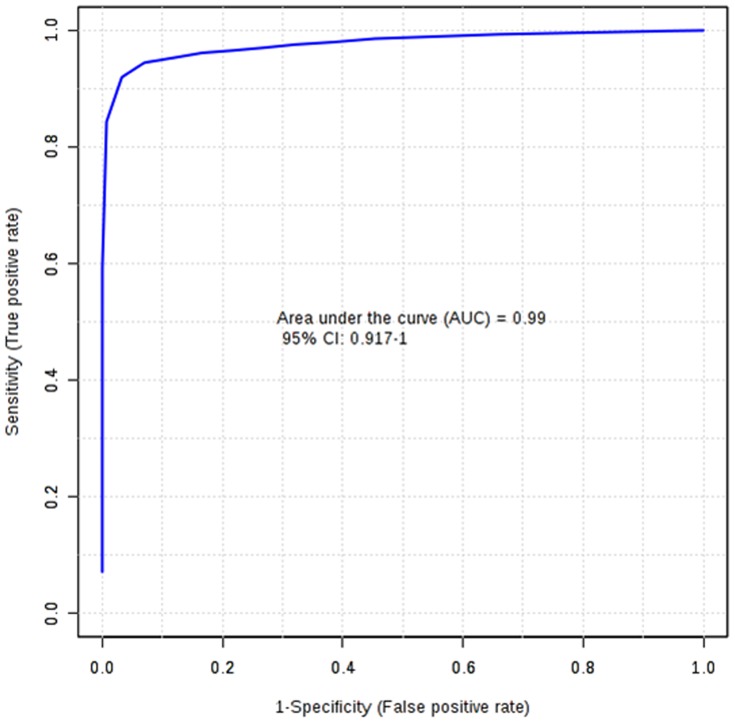
Predictive accuracy of the model discriminating carbofuran exposed and healthy control earthworms summarised using ROC curve analysis. Area under the curve = 0.99.

**Figure 7:**
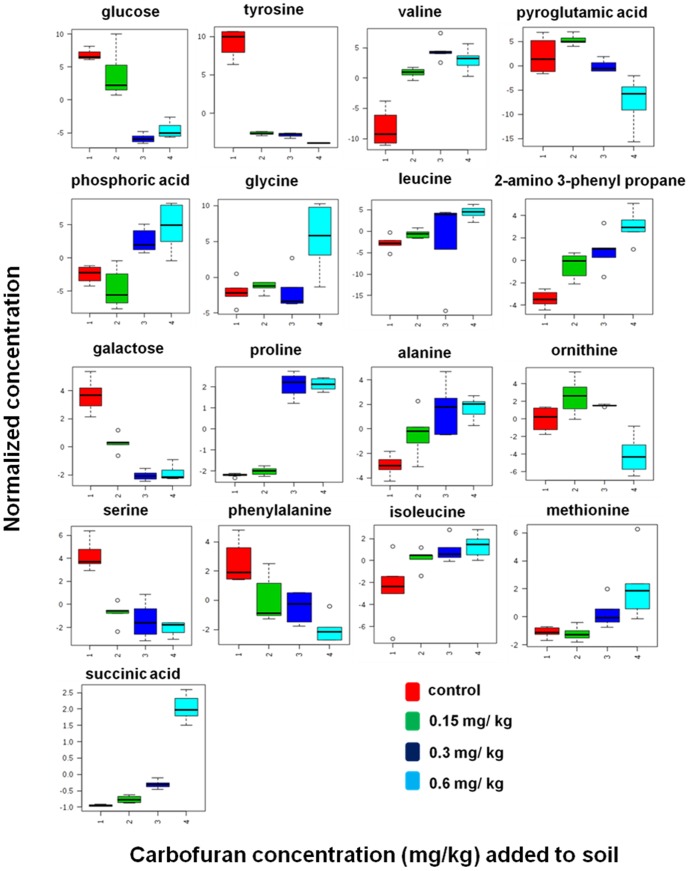
Earthworm metabolite responses to carbofuran exposure 1) control group 2) concentration of 0.15 mg kg^−1^ exposed group 3) concentration of 0.3 mg kg^−1^ exposed group 4) concentration of 0.6 mg kg^−1^ exposed group.

The potential application of the developed extraction method was evaluated to identify the metabolic perturbations of earthworms exposed to highly toxic carbamate pesticide, Carbofuran at three different concentrations (0.15, 0.3 and 0.6 mg kg^−1^). Total area normalization was performed. Initially an unsupervised PCA was applied to identify the clustering behaviour. PCA score plot has displayed the classification trend between control and carbofuran exposed samples. PCA model explained 67.0% of the total data variance with principal component 1 (PC1) explained 51.2% and principal component 2 (PC2) explained 15.8% of the total variance, respectively shown in figure 5. The PCA model with R^2^X = 0.614, Q^2^X = 0.364 has been obtained based on cumulative values up to PC 2. PLS-DA was performed to find a small number of linear combinations of the original variables (called latent variables), that was predictive for the class membership and that described most of the variability of the GC-MS metabolic profiles of control and exposed samples. Four different clusters were identified in PLS-DA score plot as depicted in Fig S4. Clear separation was identified between controls, and carbofuran exposed samples at baseline in the first PLS-DA component, suggesting that metabolomic perturbations were evident in the exposed samples. Characteristic fit criteria such as R^2^X_cum_, R^2^Y_cum_ and Q^2^Y_cum_ were examined in order to validate the PLS-DA model. R^2^X and R^2^Y represent the fraction of the variance of X matrix and Y matrix, respectively and Q^2^Y represents the predictive accuracy of the built model. The cumulative values of PLS-DA model with R^2^X_cum_ = 0.500, R^2^Y_cum_ = 0.974, Q^2^Y_cum_ = 0.968 shows good fit of the model. The supervised PLS-DA model was further validated with 500 times permutation tests (Fig S5). For this both separation distance (B/W) and prediction accuracy test statistics were performed. Permutation test statistics clearly indicated that the original class assignment is much higher compared to the B/W ratios based on permutation class assignments. The differences between the classes are statistically significant p<0.002. The magnitude of this response was higher at a concentration of 0.6 mg kg^−1^ than lower induced concentration of 0.15 mg kg^−1^. The PLS-DA model of earthworm samples was employed to explore the intrinsic differences in metabolomic profiles of control and exposed samples. Variables with VIP greater than 1 were considered to be influential for the discrimination of samples in the score plots obtained after PLS-DA experiment. Uni-variant *t*-test was performed to find out the *p*-value along with their fold change between the exposed and control samples of earthworms. Seventeen metabolites were identified as potential biomarkers among all the differential metabolites according to the VIP values (greater than 1) and *p* values less than 0.05. These metabolites belong to the classes of amino acids, carbohydrates and fatty acids. The marker metabolites along with their VIP scores and *p*-values were shown in table 2. Model sensitivity and specificity were summarised using ROC curves for the models distinguishing carbofuran exposed from healthy controls (AUROC = 0.99) Fig 6. Results were indicative of strong predictive power of the metabolite markers between Carbofuran exposed and healthy control samples. From the loading plots, the metabolites like glucose and galactose (sugars); tyrosine, valine, glycine, leucine, proline, alanine, ornithine, serine, phenyl alanine, isoleucine mehionine (amino acids) and succinic acid, pyroglutamic acid, phosphoric acid, 2-amino-3-phenyl propane (other metabolites) were showed significant fluctuations in response to carbofuran exposure. PC1, which was shown to explain the variation due to exposure concentration, was influenced more by amino acids and sugars. The relative concentration changes in discernible metabolite intensities were examined to determine the fluctuations in the metabolites of earthworms after their exposure to carbofuran as depicted in fig 7. Sugars like glucose and galactose were shown significant decrease in their concentration levels in carobofuran exposed earthworms in comparison to controls. This decrease was suggested to be as a result of increase in the energy requirements of earthworms exposed to carbofuran. The decrease was more in the concentrations of 0.3 mg/kg and 0.6 mg/kg in comparison to 0.15 mg/kg. This observation may also be a result of energy metabolism being disturbed by the toxicity of carbofuran at high exposure concentrations.

**Table 2:** Key putatively identified metabolic perturbations from GC MS based metabolomic analyses of earthworms after exposed to carbofuran.

S.no	Marker metabolite	p-value	VIP	fold change
1	Glucose	2.1518E-10	3.20	decreased
2	Tyrosine	1.4389E-16	2.89	decreased
3	Valine	8.8602E-10	2.77	increased
4	Pyroglutamic acid	1.0541 E-5	2.39	decreased
5	Phosphoric acid	4.0752E-6	2.10	increased
6	Glycine	1.3342E-4	1.62	increased
7	Leucine	2.0883E-8	1.54	increased
8	2-amino,3-phenyl propane	1.4289E-7	1.51	increased
9	Galactose	1.127E-11	1.39	decreased
10	Proline	3.8104E-17	1.29	increased
11	Alanine	3.5999E-5	1.26	increased
12	Ornithine	2.4149E-6	1.21	decreased
13	Serine	1.1611E-7	1.19	decreased
14	Phenyl alanine	3.4904E-4	1.16	decreased
15	Isoleucine	1.6252E-5	1.12	increased
16	Methionine	1.5858E-4	1.06	increased
17	Succinic acid	4.7329E-16	1.04	increased

Increase in levels of alanine is an universal indicator for stress in various organisms exposed to different external contaminants [74]. In the present study, alanine was significantly increased in earthworms exposed to carbofuran. The increased response of alanine after carbofuran exposure was observed with increase in the dose of carbofuran from 0.15 mg/kg to 0.6 mg/kg indicates that alanine may be an indicator for carbofuran exposure. Succinate, a Krebs cycle intermediate showed a significant increase in carbofuran exposed earthworms. It may be due to possible disruption in the succinate dehydrogenase enzyme function [75]. Succinate dehydrogenase is the only enzyme of the Krebs cycle that is bound to inner mitochondrial membrane [75]. Five amino acids such as tyrosine, pyroglutamic acid, ornithine, serine and phenyl alanine were down regulated in carbofuran exposed earthworms in comparison to control. Seven amino acids valine, glycine, leucine, proline, alanine, isoleucine, methionine were up-regulated in carbofuran exposed earthworms in comparison to control. The present study indicated that amino acid and carbohydrate metabolism were mainly disturbed in carbofuran exposed earthworms. The present results were well in agreement with earlier reports on fish exposure to carbofuran, where the disturbances were shown to occur in protein and carbohydrate metabolism [76].

## Conclusion

Environmental metabolomics is a rapidly developing and emerging sub-discipline of metabolomics and has the potential to relate between earthworm toxicity and bioavailability of soil contaminants. It necessitates the need for simple and robust methods with precise and accurate extraction of metabolites from earthworms. As the sample preparation is crucial for the metabolomic profiling of earthworms, extraction conditions for non targeted metabolomic approach was first optimized. Then, we applied the nontargeted metabolomic approach to identify the biomarkers of carbofuran induced toxicity in earthworm, *Metaphire posthuma* with the aid of multivariate analysis. Earthworms exhibited significant perturbations in their metabolomic profiles after their exposure to carbofuran. This study suggests that, the high sensitivity, specificity and availability of spectral libraries of mass spectrometer combined with gas chromatography having good resolution ability, makes the gas chromatography-mass spectrometry an excellent tool for metabolomics application to identify the biomarkers for environmental toxicants and contaminants exposure.

## Supporting Information

Figure S1 **Yields of the identified metabolites.** A) Amino acids B) Carbohydrates C) Fatty acids D) Organic acids E) Phosphates F) Polyols.
(TIF)

Figure S2 **PCA bi plot for solvent systems (80% MeOH, MIPW, Pure MeOH, AMW, MCW) and extracted metabolites.** Bi plot clearly indicate most of the metabolites clustered together with 80% Methanol, 100% MeOH, MIPW.
(TIF)

Figure S3 **GC-MS chromatogram for extracted metabolites using different solvent systems include, 80% MeOH, MIPW, Pure MeOH, AMW, MCW.**
(TIF)

Figure S4 **PLS-DA scores plot for 1) control earthworms and earthworms exposed to soils spiked with 2) 0.15 mg/kg 3) 0.3 mg/kg 4) 0.6 mg/kg.**
(TIF)

Figure S5 **Validation results.** A) screen shot showing a PLS-DA cross validation. B) Permutation analysis of PLS-DA models derived from carbofuran exposed and healthy controls. Statistical validation of the PLS-DA by permutation analysis using 500 different model permutations. The goodness of fit and predictive capability of the original class assignments is much higher compared to ratios based on the permutation class assignments.
(TIF)

Table S1 **Total number of peaks in earthworm** ***Metaphire posthuma*** **.**
(DOC)
