# Supplementary figures and images for: Gas Chromatography- Mass Spectrometry Based Metabolomic Approach for Optimization and Toxicity Evaluation of Earthworm Sub-Lethal Responses to Carbofuran
Source: PLoS One. 2013 Dec 4;8(12):e81077. doi: 10.1371/journal.pone.0081077 (PMC3852017; doi:10.1371/journal.pone.0081077)

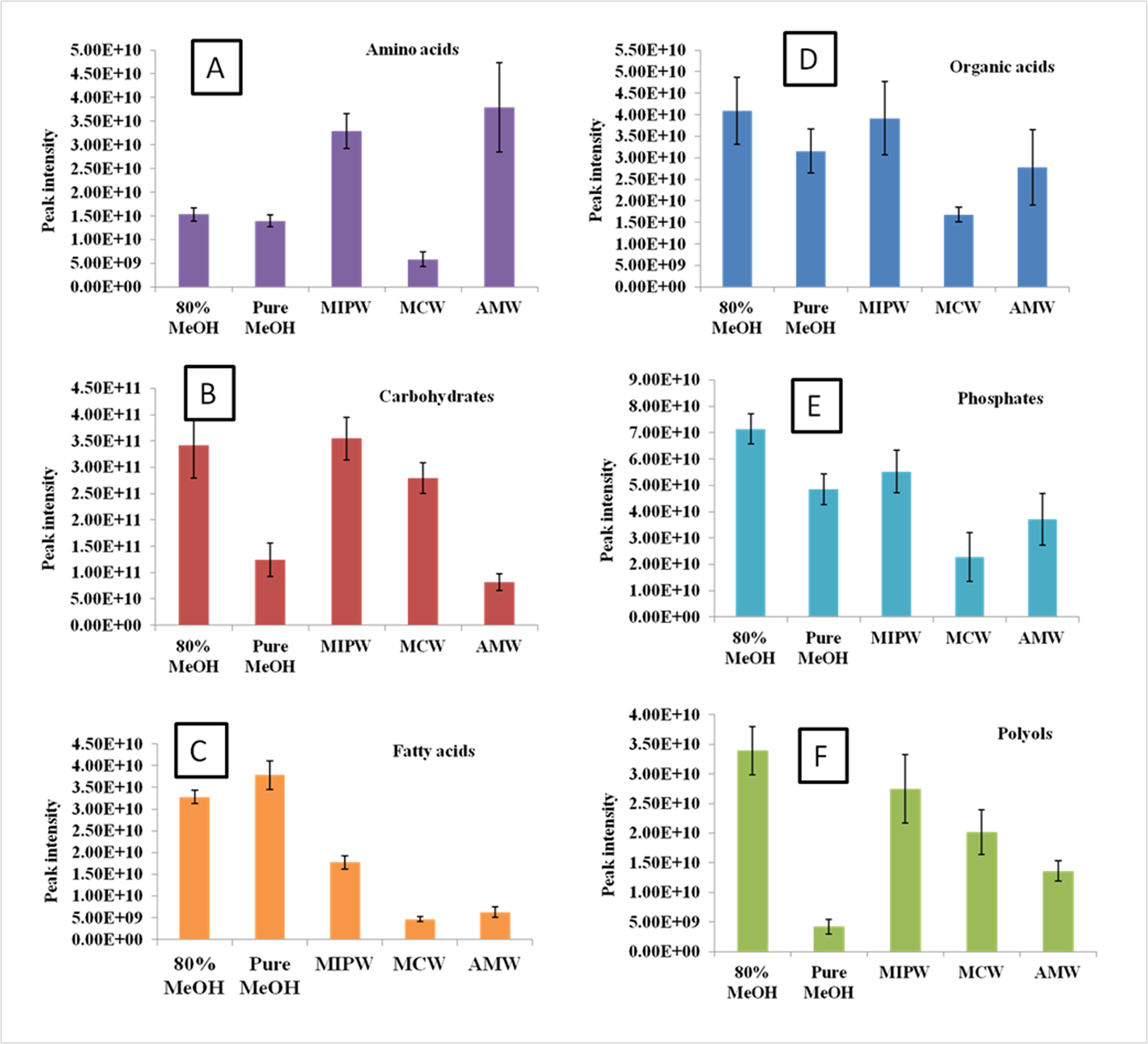

Supplement: Figure S1 — Yields of the identified metabolites. A) Amino acids B) Carbohydrates C) Fatty acids D) Organic acids E) Phosphates F) Polyols. (TIF) [file pone.0081077.s001.tif]

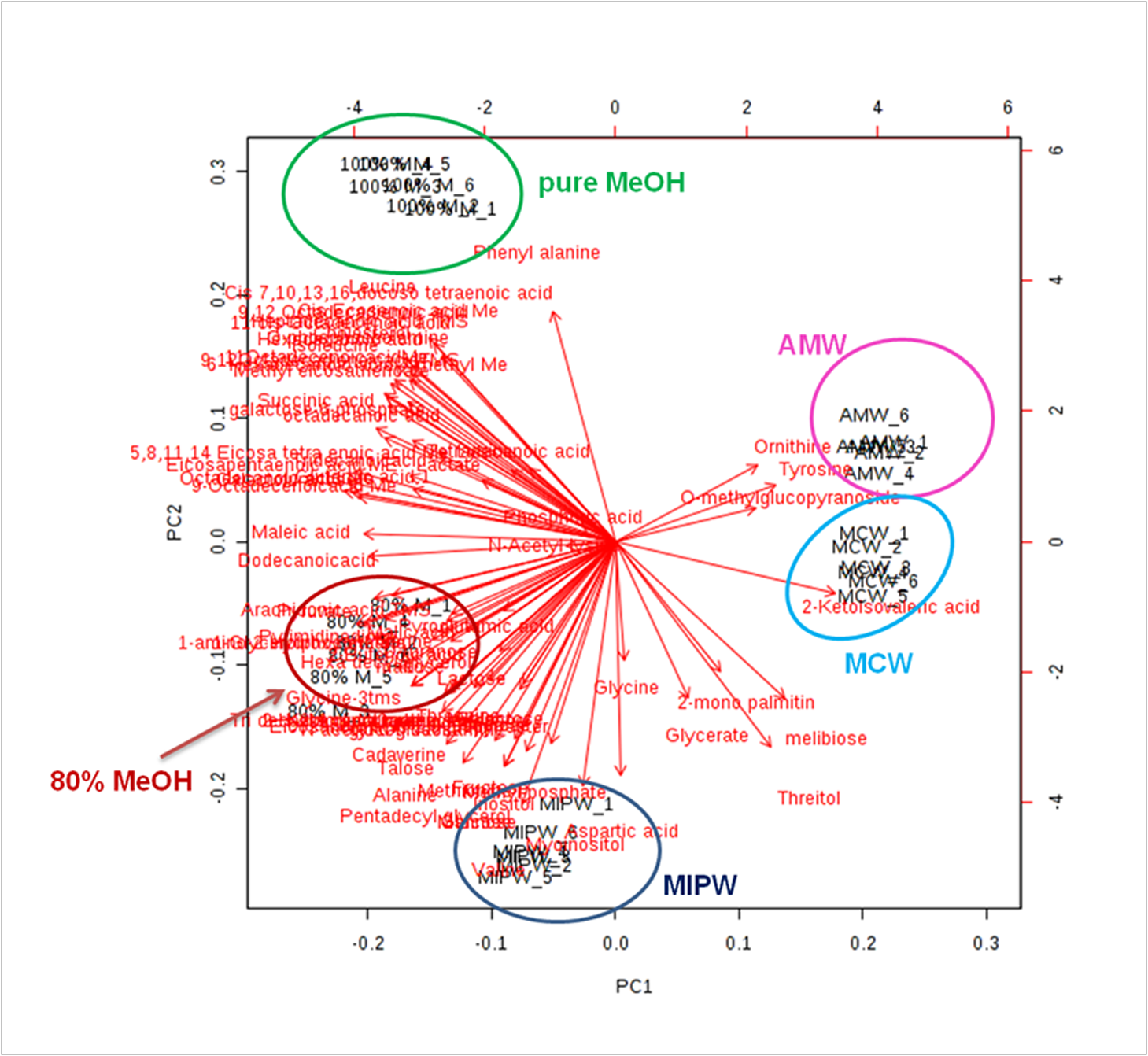

Supplement: Figure S2 — PCA bi plot for solvent systems (80% MeOH, MIPW, Pure MeOH, AMW, MCW) and extracted metabolites. Bi plot clearly indicate most of the metabolites clustered together with 80% Methanol, 100% MeOH, MIPW. (TIF) [file pone.0081077.s002.tif]

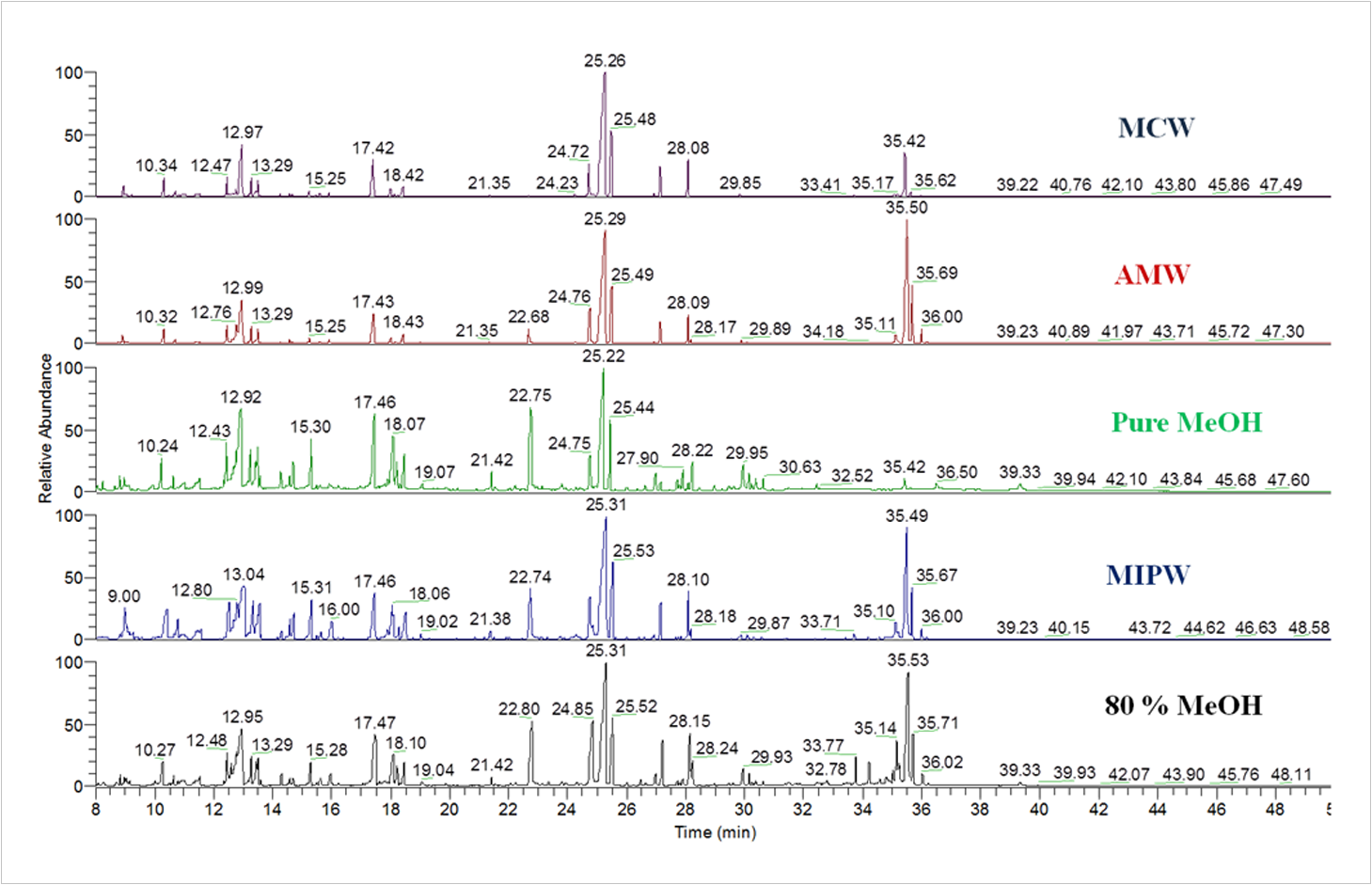

Supplement: Figure S3 — GC-MS chromatogram for extracted metabolites using different solvent systems include, 80% MeOH, MIPW, Pure MeOH, AMW, MCW. (TIF) [file pone.0081077.s003.tif]

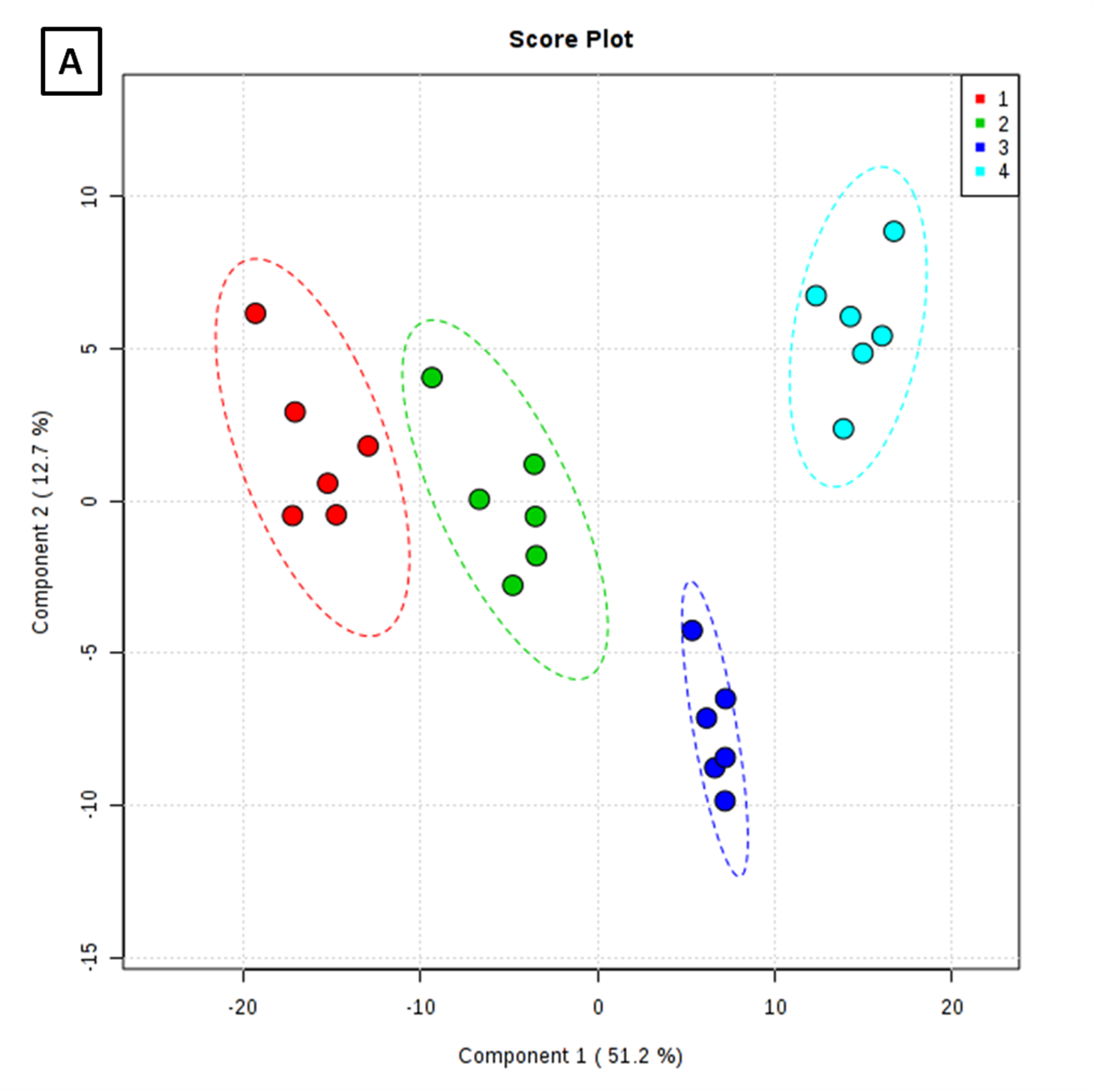

Supplement: Figure S4 — PLS-DA scores plot for 1) control earthworms and earthworms exposed to soils spiked with 2) 0.15 mg/kg 3) 0.3 mg/kg 4) 0.6 mg/kg. (TIF) [file pone.0081077.s004.tif]

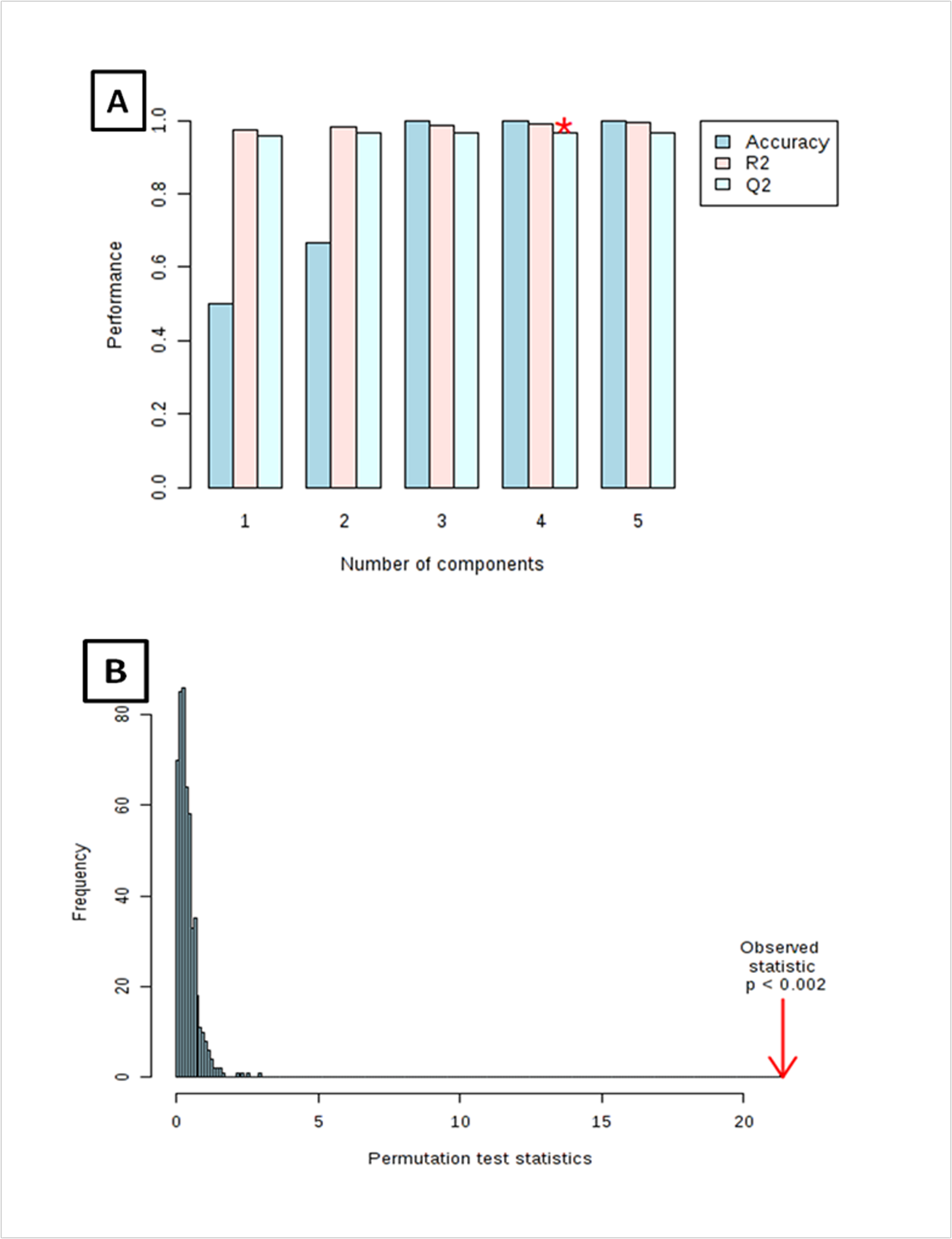

Supplement: Figure S5 — Validation results. A) screen shot showing a PLS-DA cross validation. B) Permutation analysis of PLS-DA models derived from carbofuran exposed and healthy controls. Statistical validation of the PLS-DA by permutation analysis using 500 different model permutations. The goodness of fit and predictive capability of the original class assignments is much higher compared to ratios based on the permutation class assignments. (TIF) [file pone.0081077.s005.tif]
