# Supplementary material for: Gas Chromatography- Mass Spectrometry Based Metabolomic Approach for Optimization and Toxicity Evaluation of Earthworm Sub-Lethal Responses to Carbofuran
Source: PLoS One. 2013 Dec 4;8(12):e81077. doi: 10.1371/journal.pone.0081077 (PMC3852017; doi:10.1371/journal.pone.0081077)
Supplement: Table S1 — Total number of peaks in earthworm Metaphire posthuma . (DOC) [file pone.0081077.s006.doc]

**Table S 1. Total number of peaks in earthworm *Metaphire posthuma***

| Peaks | Number of peaks | | | | |
| --- | --- | --- | --- | --- | --- |
|  | Pure MeOH | AMW | 80% MeOH | MCW | MIIPW |
| Identified | 84.6±3.1 | 88.8±2.0 | 91.2±2.4 | 86.2±2.5 | 89.8±3.3 |
| Unidentified | 136.7±15.8 | 224.0±15.9 | 233.5±10.8 | 150.5±20.6 | 227.5±13.8 |
| Total | 221.5±16.4 | 312.8±17.6 | 324.8±12.1 | 236.8±21.2 | 317.3±15.2 |
